# The genetics of a “femaleness/maleness” score in cardiometabolic traits in the UK biobank

**DOI:** 10.1038/s41598-023-36132-1

**Published:** 2023-06-05

**Authors:** Daniel E. Vosberg, Zdenka Pausova, Tomáš Paus

**Affiliations:** 1 Centre Hospitalier Universitaire Sainte-Justine, University of Montreal, Montreal, QC Canada; 2 Department of Neuroscience, Faculty of Medicine, University of Montreal, Montreal, QC Canada; 3 Research Institute of the Hospital for Sick Children, Toronto, ON Canada; 4 Departments of Physiology and Nutritional Sciences, University of Toronto, Toronto, ON Canada; 5 Departments of Psychology and Psychiatry, University of Toronto, Toronto, ON Canada; 6 ECOGENE-21, Chicoutimi, QC Canada; 7 Department of Psychiatry and Addictology, Faculty of Medicine, University of Montreal, Montreal, QC Canada

**Keywords:** Genetic association study, Genetics of the nervous system, Obesity

## Abstract

We recently devised continuous “sex-scores” that sum up multiple quantitative traits, weighted by their respective sex-difference effect sizes, as an approach to estimating polyphenotypic “maleness/femaleness” within each binary sex. To identify the genetic architecture underlying these sex-scores, we conducted sex-specific genome-wide association studies (GWASs) in the UK Biobank cohort (females: n = 161,906; males: n = 141,980). As a control, we also conducted GWASs of sex-specific “sum-scores”, simply aggregating the same traits, without weighting by sex differences. Among GWAS-identified genes, while sum-score genes were enriched for genes differentially expressed in the liver in both sexes, sex-score genes were enriched for genes differentially expressed in the cervix and across brain tissues, particularly for females. We then considered single nucleotide polymorphisms with significantly different effects (sdSNPs) between the sexes for sex-scores and sum-scores, mapping to male-dominant and female-dominant genes. Here, we identified brain-related enrichment for sex-scores, especially for male-dominant genes; these findings were present but weaker for sum-scores. Genetic correlation analyses of sex-biased diseases indicated that both sex-scores and sum-scores were associated with cardiometabolic, immune, and psychiatric disorders.

## Introduction

In animals, including humans, there are numerous sex differences that extend well beyond sex hormones and reproductive systems. Sex differences in multiple physiological, developmental, and behavioural traits have been delineated in species ranging from Drosophila melanogaster^1^ to cetaceans^2^. In a study of 14,250 wildtype mice, over half (56.6%) of the 903 datasets, comprising 225 continuous traits, demonstrated sex differences^3^. Conserved sex-bias in gene expression has been identified in an investigation of five mammalian species (human, macaque, mouse, rat, and dog) across 12 tissues^4^. Moreover, in wild mammals (101 species), the median life expectancy is 18.6% longer among females, as compared with males, thus indicating the relevance of sex differences for morbidity and mortality^5^.

In humans, sex differences are evident in many continuous traits. For example, adult females (vs. males) have a higher fat mass, lower lean-body mass, and preferentially deposit fat subcutaneously, while males (vs. females) have a greater amount of visceral fat^6,7^. Perhaps not surprisingly, there are sex differences in the prevalence, expression, and outcomes of physical and mental disorders. In the United States, for example, there are subtle albeit significant differences in the percentages of each sex who die of heart disease (females: 21.8%; males: 24.2%), cancer (females: 20.7%; males: 21.9%), stroke (females: 6.2%; males: 4.3%), type 2 diabetes (females: 2.7%; males: 3.2%), and Alzheimer’s disease (females: 6.1%; males: 2.6%)^8,9^. The prevalence of autoimmune, chronic pain, eating, and anxiety disorders is higher in females while the opposite is true about Parkinson's Disease, autism, attention-deficit hyperactivity disorder, and oppositional defiant disorder^8,10,11^. These phenotypic sex differences likely stem from both genetic and environmental (including socio-cultural) influences^8,12,13^. For instance, eating disorders and depression may be underdiagnosed in men due to sociocultural influences^14,15^.

At a molecular level, investigators recently delineated genetic sex-differences across complex traits in ~ 450,000 middle-aged adults in the UK Biobank^16^. Among the ~ 84 continuous phenotypes, there were (i) sex differences in heritability for 48.88% of traits, (ii) inter-sex genetic correlations lower than r_g_ = 1 in 69.88% of traits indicating a global deviation between the sexes in the genetic effects on a given trait, and (iii) significant sex differences (in the strength/direction of genotype–phenotype associations) for at least one autosomal single nucleotide polymorphism (SNP) for 72.62% of traits^16^. The largest number of sex-different SNPs were identified for anthropometric traits including the ratio of waist-to-hip circumference, standing height, and trunk fat-percentage^16^.

While many sex differences in continuous traits are undoubtedly robust, the distributions for a given trait of each sex almost invariably overlap. Thus, our group recently devised continuous polyphenotypic “sex-scores” that capture, within each sex, "femaleness/maleness", by summing up standardized values across quantitative traits, weighted by respective sex-difference effect-sizes^17^. We use the term “femaleness/maleness” rather than “masculinity/femininity" since our sex-scores are based on quantitative sex differences (i.e., females vs. males) rather than self-reported measures of conformity to gender roles or stereotypes. The initial study of these sex-scores, carried out in a community-based sample of adolescents, revealed within-sex correlations of several traits (e.g., testosterone, externalizing behaviour) with the individual’s “femaleness/maleness”, thus complementing a binary biological (male vs. female) approach to the study of sex differences^17^.

In the current report, our first aim was to elucidate the molecular architecture underlying sex-scores based on routinely assessed anthropometric and metabolic phenotypes. To tease apart whether our genetic findings are driven by latent “femaleness/maleness” or the simple aggregation of traits, we also evaluated the genetic architecture underlying “sum-scores”, whereby we summed up the standardized traits, without applying a sex-difference weighting. Thus, we performed sex-specific genome-wide association studies (GWAS) in the UK Biobank of sex-scores and sum-scores. Our second aim was to investigate the genetic correlations among the scores between the two sexes and sex differences in these scores at the level of SNPs (“sex different” SNPs [sdSNPs]). Next, we assessed genetic correlations between sex-scores and sum-scores and clinical conditions with a sex-biased prevalence. Finally, we assessed the degree of pleiotropy among sex-score SNPs and sum-score SNPs, to estimate the extent to which the SNPs were capturing variance across the composite traits.

## Results

### Polyphenotypic sex-scores and sum-scores

**Figure 1:**
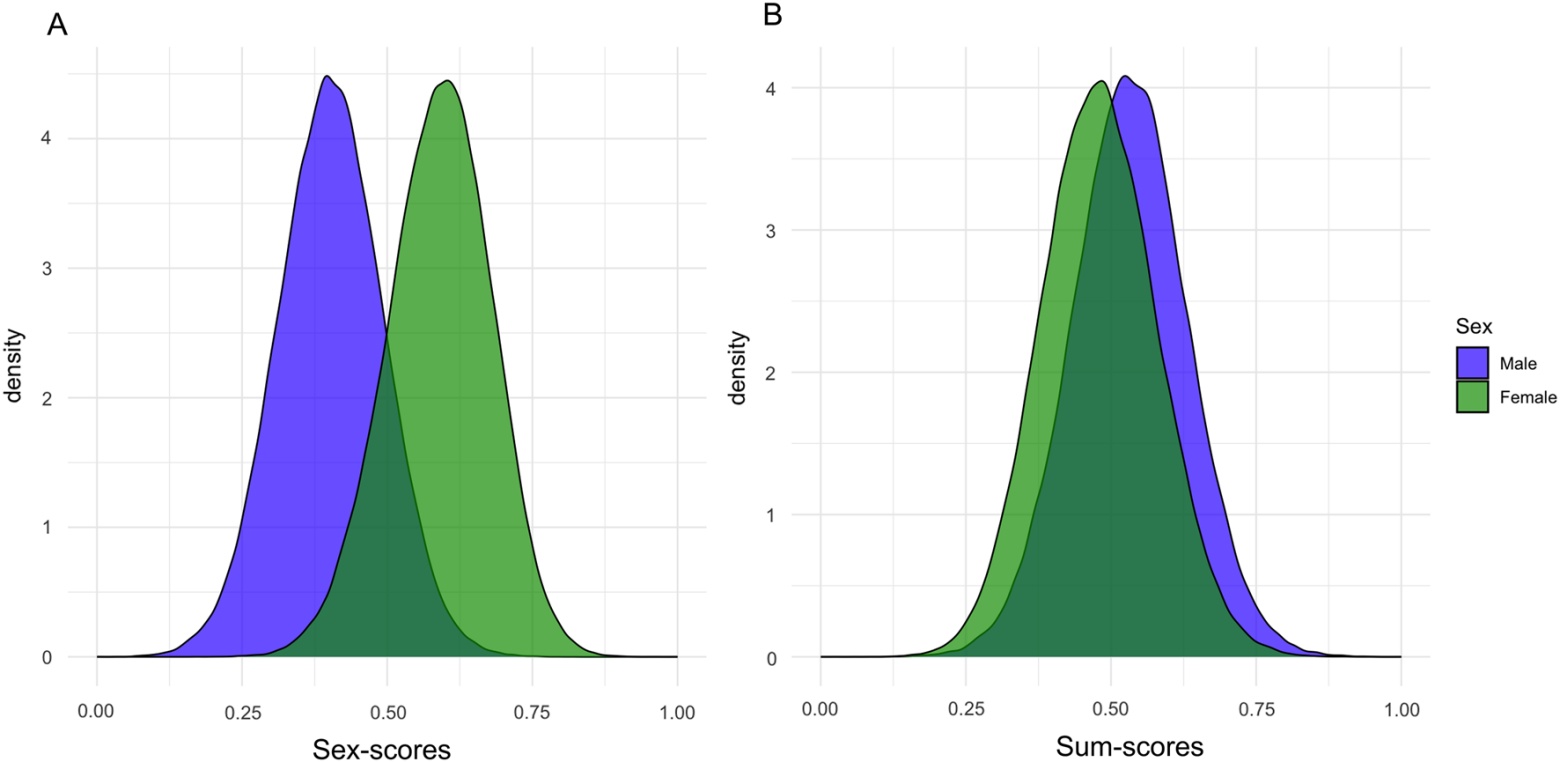
Distributions of sex-scores and sum-scores. Density plots for the distributions of age-adjusted (**A**) sex-scores and (**B**) sum-scores, which overlap between the sexes. Blue indicates males and green indicates females. Whereas, by design, higher sex-scores indicate higher "femaleness" and are higher among females, compared with males (Cohen’s D = 2.08, t_297,788_ = 572.17, *p* < 1e−300), the sum-scores are subtly higher among males (Cohen’s D = 0.53, t_301,443_ = 147.44, *p* < 1e−300).

To compute sex-scores, we first selected 13 commonly assessed anthropometric and cardiometabolic traits in the UK Biobank (Fig. S1). Each of these were assessed in at least 100,000 participants and were available in other cohorts including, for example, the Saguenay Youth Study (SYS), the Cardiovascular Health Study (CHS), the Framingham Heart Study (FHS) and the Rotterdam study (RS)^18–21^. To adjust for correlations among the comprising traits, pairs of traits with correlations exceeding a threshold (r^2^ = 0.25) were averaged (Fig. S2); body mass index (BMI) was not included as it is a mathematical combination of weight and height. Next, we computed sex-scores by summing up standardized values across traits, each weighted by respective sex-difference effect sizes, and adjusted for age at recruitment (Table 1). Note that, by design, higher sex-scores indicate higher “femaleness” (in both sexes; Fig. 1A). Additionally, we computed “sum-scores” by summing up standardized values across traits per individual, *without* weighting by the sex-difference effect sizes (Fig. 1B). Confirming that the variability in sex-scores was not entirely determined by the aggregation of traits, the sum-scores were phenotypically correlated with sex-scores but explained a fraction of the variance (males: r =  − 0.37, r^2^ = 0.14, *p* < 1 × 10^–300^; females: r =  − 0.44, r^2^ = 0.19, *p* < 1 × 10^–300^).

**Table 1:** Age-adjusted sex difference effect sizes.

Domain	Phenotype	N (Females)	N (Males)	Effect size (F > M)	*p* value
Biochemistry	HDL-cholesterol	69,422	56,440	0.84	< 1 × 10^–300^
Biochemistry	Average of cholesterol and LDL-cholesterol	92,110	72,251	0.27	< 1 × 10^–300^
Cardiovascular	Pulse rate	92,466	71,491	0.15	9.76 × 10^–208^
Biochemistry	CRP	92,224	72,376	0.06	6.92 × 10^–31^
Biochemistry	Glucose	68,602	55,129	− 0.12	4.95 × 10^–104^
Cardiovascular	Average of diastolic and systolic blood pressure	86,120	66,764	− 0.31	< 1 × 10^–300^
Biochemistry	Triglycerides	92,524	72,722	− 0.43	< 1 × 10^–300^
Anthropometric	Average of weight and waist circumference	109,966	85,590	− 0.92	< 1 × 10^–300^
Anthropometric	Height	110,196	85,684	− 1.40	< 1 × 10^–300^

Age-adjusted sex difference effect sizes (betas) across routinely assessed cardiometabolic and anthropometric traits, in the UK biobank cohort. Due to the moderate correlations (r > 0.5) among certain trait pairs, we averaged (1) cholesterol and LDL, (2) systolic and diastolic blood pressure, and (3) weight and waist circumference. Note that the samples used to compute effect sizes are independent from those used for the GWASs. The *p*-values of “ < 1 × 10^–300^” indicate values that are below R’s floating-point number limit.

### Genome wide association study (GWAS) of sex-scores and sum-scores

**Figure 2:**
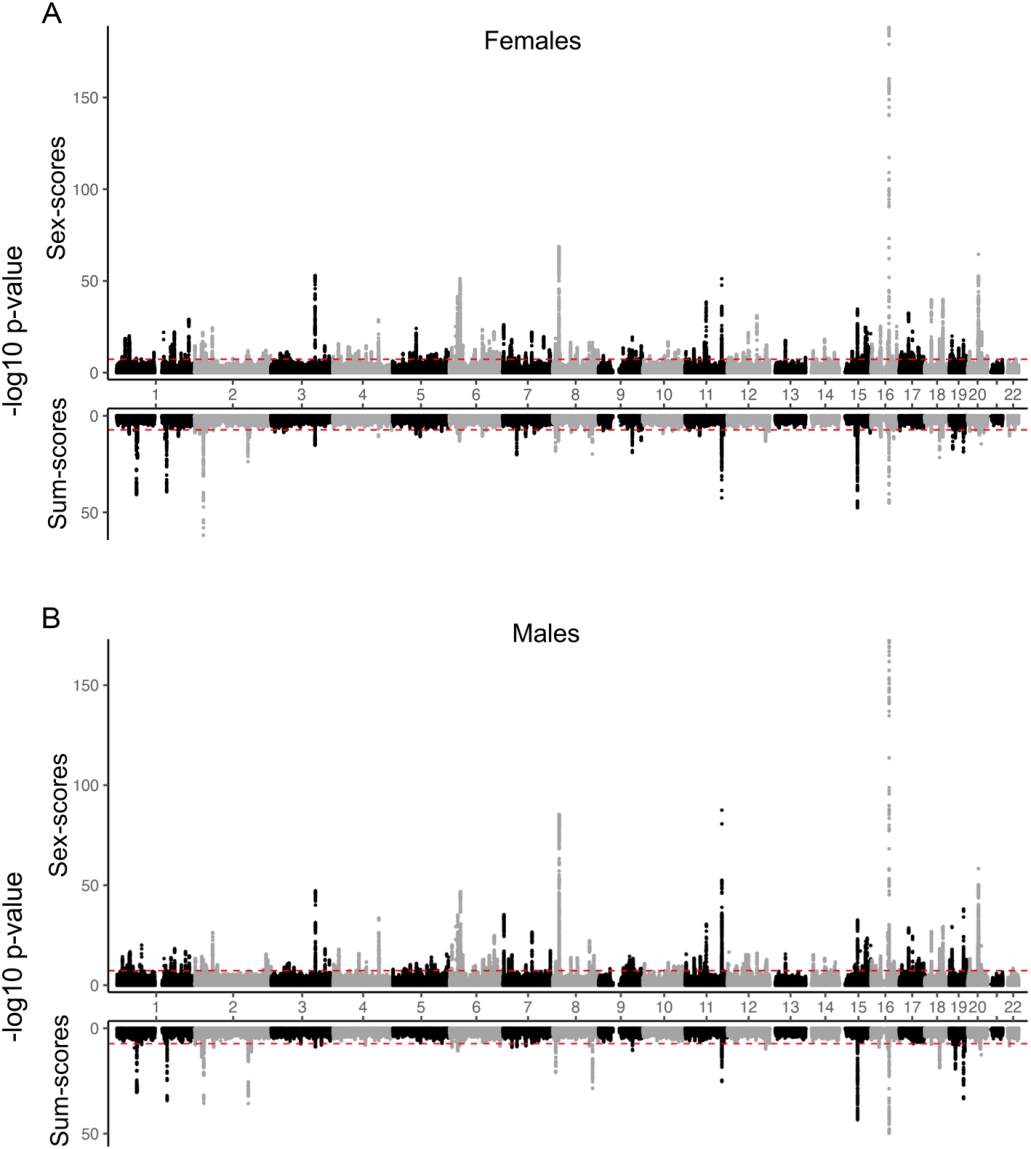
Sex-score and sum-score Miami plot in (**A**) females and (**B**) males. The horizontal red dotted bars indicate the thresholds for genome-wide statistical significance. For females (n = 161,906), there were 1373 GWAS-sig. SNPs (1242 genes) for sex-scores, and 331 GWAS-sig. SNPs (317 genes) for sum-scores. For males (n = 141,980), sex-scores, there were 1227 GWAS-sig. SNPs (1110 genes) for sex-scores, and 216 GWAS-sig. SNPs (180 genes) for sum-scores.

**Figure 3:**
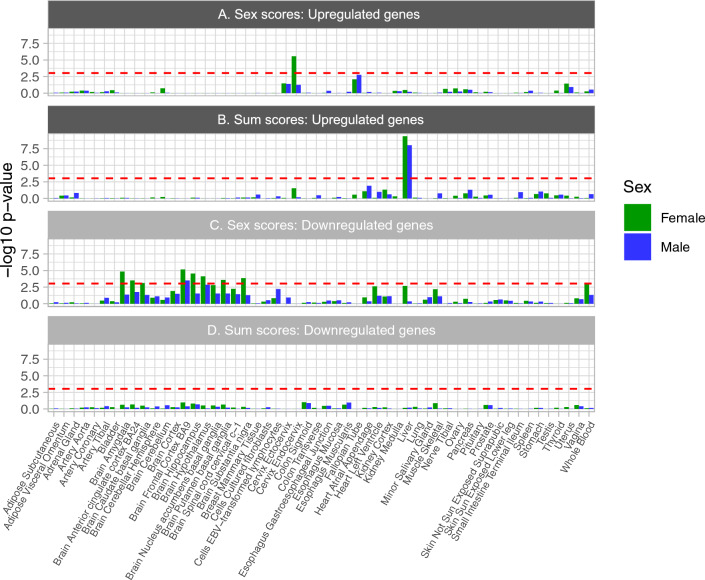
Gene enrichment of GWAS genes in GTEx v8 with 54 tissues. The horizontal red bars indicate statistically significant findings, surviving a Bonferroni correction for 54 tissues (*p* = 0.00093). A hypergeometric test was conducted to assess enrichment of genes for differentially expressed genes (DEG) sets. The Y-axis indicates the enrichment p-value for the intersect between upregulated DEG and (**A**) sex-scores and (**B**) sum-scores, and downregulated DEG for (**C**) sex-scores and (**D**) sum-scores. The DEGs were determined by assessing standardized, log2-transformed gene expression (transcript per million [TPM] or reads per kilobase of transcript per million mapped reads [RPKM]) in one region, versus all the other regions (absolute log fold change ≥ 0.58; *p*_bonferroni_ ≤ 0.05)^22^.

To elucidate the genetic architecture underlying polyphenotypic sex-scores, we conducted sex-specific genome-wide association studies (GWASs). The results of these two GWASs are presented in the Miami plots in Fig. 2. Following the GWASs, we used FUMA-GWAS^22^ for positional mapping of SNPs to genes and for assessing the function of these genes. For sex-scores, we identified 1373 independent genome-wide significant SNPs (GWAS-sig. SNPs), mapping to 1242 genes in females (n = 161,906) and 1227 GWAS-sig. SNPs (1110 genes) in males (n = 141,980). In comparison, for sum-scores, there were 331 GWAS-sig. SNPs (317 genes) in females and 216 GWAS-sig. SNPs (180 genes) in males (Tables S1-2). We conducted enrichment analyses using ‘GENE2FUNC’ with the FUMA-GWAS platform, identifying enrichment for numerous Gene Ontology Biological Processes (GO-BP) for sex-scores (females: 249 terms; males: 161 terms) and sum-scores (females: 136 terms; males: 157 terms; Tables S3A-D). For sex-scores, but not sum-scores, these included hormone-related terms for females (e.g., “cellular response to peptide hormone stimulus”, “steroid hormone mediated signalling pathway”, “cellular response to growth hormone stimulus”) and males (e.g., “cellular response to growth hormone stimulus”, “response to growth hormone”). To assess systematically the most prominent overall similarities and differences in GO-BP enrichment patterns between sex-scores and sum-scores, we used R’s ‘clusterProfiler’^23^. Here, we identified that the top enrichment terms were implicated in chromatin, protein-lipid remodelling, and homeostasis of lipids, triglycerides, and cholesterol and these were significant and highly similar across all four GWASs, with subtle variations in the effect sizes (Fig. S3−4). Nevertheless, striking differences emerged between the sex-scores and sum-scores GWASs in a Genotype-Tissue Expression (GTEx) v8 54 tissue analysis, using FUMA. Namely, while female sex-score genes were enriched for the upregulated ‘cervix/endocervix’ gene set, they were downregulated for numerous brain-tissue gene sets including the frontal cortex, amygdala, hippocampus, hypothalamus, substantia nigra, putamen, anterior cingulate cortex, and caudate nucleus. In comparison, male sex-score genes were only enriched for the downregulated frontal-cortex gene set, with nominally significant effects among other brain tissues. By contrast, sum-scores genes for both sexes were strongly enriched for genes upregulated in the liver (Fig. 3).

### Genetic correlations and SNP-based heritability of the sex- and sum-scores

Next, we conducted genetic correlations between the two sexes for each score and between the two scores for each sex, using linkage disequilibrium score regression (LDSC) version 1.0.1^24,25^. While the between-sex genetic correlations were high for sex-scores (r_g_ = 0.95, SE = 0.012, *p* < 1 × 10^–300^) and sum-scores (r_g_ = 0.91, SE = 0.02, *p* < 1 × 10^–300^), both differed significantly from 1 (sex-scores: z = 4.44, *p* = 9.08 × 10^–6^; sum-scores: z = 3.71, *p* = 2.07 × 10^–4^). Moreover, the genetic correlations between sex-scores and sum-scores were moderate among females (r_g_ =  − 0.57, SE = 0.028, *p* = 4.77 × 10^–91^) and males (r_g_ =  − 0.53, SE = 0.03, *p* = 2.90 × 10^–62^). Additionally, the SNP-based heritabilities, estimated by LDSC, were notably higher for sex-scores (female h^2^ = 0.294; male h^2^ = 0.308), relative to sum-scores (female h^2^ = 0.155; male h^2^ = 0.128).

### Sex-different single nucleotide polymorphisms (sdSNPs)

**Figure 4:**
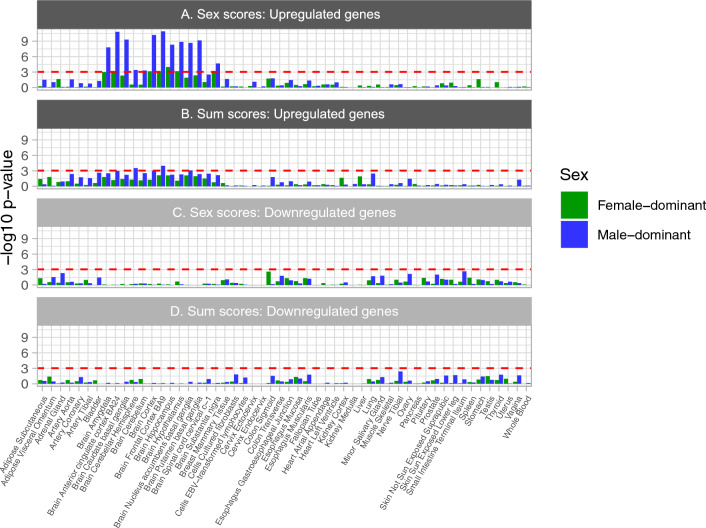
Gene enrichment of Sex-different SNP genes in GTEx v8 with 54 tissues. The red bars indicate statistically significant findings, surviving a Bonferroni correction. A hypergeometric test was conducted to assess enrichment of genes for differentially expressed genes (DEG) sets. The Y-axis indicates the enrichment p-value for the intersect between upregulated DEG and (**A**) sex-scores and (**B**) sum-scores, and downregulated DEG for (**C**) sex-scores and (**D**) sum-scores. The DEGs were determined by assessing standardized, log2-transformed gene expression (transcript per million [TPM] or reads per kilobase of transcript per million mapped reads [RPKM]) in one region, versus all the other regions (absolute log fold change ≥ 0.58; *p*_bonferroni_ ≤ 0.05)^22^.

At a fine-grained level of sex-score genetics, we identified 9,997 “female-dominant” sdSNPs and 13,422 “male-dominant” sdSNPs (see Methods for definition of “dominant”), at a *p*-value threshold of 1 × 10^–5^, and 776 female-dominant sdSNPs and 836 male-dominant sdSNPs at a threshold of *p* < 5 × 10^–8^ (Table S4A, B). Using MAGMA, we identified 162 female-dominant genes and 216 male-dominant genes in males that survived a gene-wide adjustment in each sex (*p* < 2.99 × 10^–6^; *p* = 0.05/16,710 genes in MAGMA; Table S5). Note that only 6 genes (*FHIT*, *CSMD1*, *PTPRD*, *RBFOX1*, *WWOX*, and *CDH13*)*,* were found in common between the sexes; these were excluded in the subsequent analysis. For sum-scores, we identified 1761 female-dominant sdSNPs and 2,708 male-dominant sdSNPs at a *p*-value threshold of 1 × 10^–5^, and 38 female-dominant sdSNPs and 71 male-dominant sdSNPs at a threshold of *p* < 5 × 10^–8^ (Table S4C, D). Using MAGMA, we identified 42 female-dominant genes and 86 male-dominant genes in males that survived a gene-wide adjustment in each sex (*p* < 3.11 × 10^–6^; *p* = 0.05/16,069 genes in MAGMA; Table S5). Two genes, *CDH18* and *WWOX,* intersected between the sexes and were excluded in the subsequent analysis. Conducting a GTEx analysis with FUMA for these sex-different genes, the male-dominant sex-score genes were enriched for genes upregulated across 12 brain tissues, namely the frontal cortex, anterior cingulate cortex, brain cortex, caudate nucleus, basal ganglia, hypothalamus, nucleus accumbens, hippocampus, amygdala, substantia nigra, cerebellar hemisphere, and cerebellum, all surviving a Bonferroni correction. By contrast, the female-dominant sex-score genes were enriched for genes differentially expressed in the hypothalamus, hippocampus, frontal cortex, and cortex, all surviving a Bonferroni correction. The male-dominant sum-score genes were enriched for genes differentially expressed in the frontal cortex, cerebellar hemisphere, and nucleus accumbens, while there was no enrichment of female-dominant sum-score genes in differentially expressed gene sets (Fig. 4).

### Genetic correlations with the comprising traits and disorders

**Figure 5:**
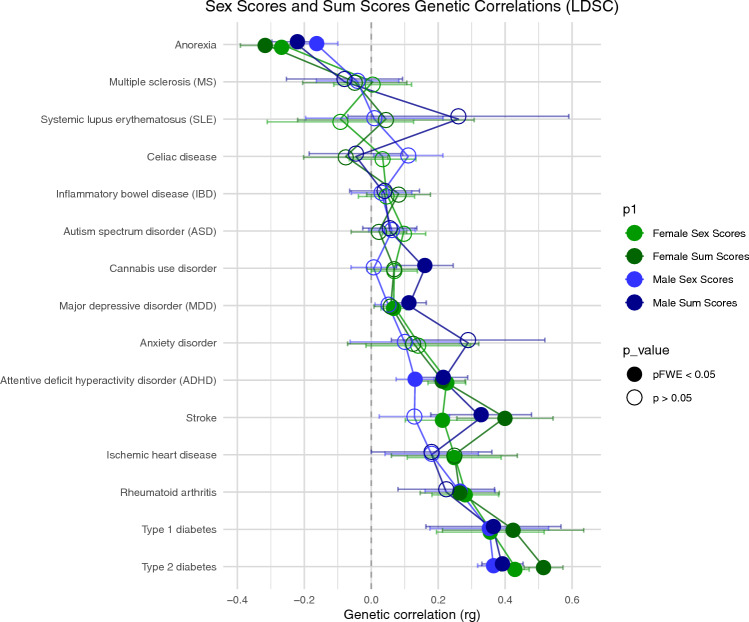
Genetic correlations of the sex-scores and sum-scores with sex-biased and cardiometabolic disorders. To facilitate comparisons between scores, the sign of sex-scores was flipped. Generated using linkage disequilibrium score regression (LDSC), sex-stratified genetic correlations were conducted between the sex-score and sum-score GWASs and sex-biased disorder GWASs. The significant effects are filled-in, surviving a Bonferroni correction for 60 genetic correlations for each score (15 traits × 2 sexes × 2 scores; *p* < 0.00083). The error bars represent the 95% confidence intervals.

We found positive genetic correlations (i.e. higher femaleness, higher trait values) between the sex-specific sex-scores and HDL-cholesterol, total cholesterol, and LDL-cholesterol (males only), and negative genetic correlations between the sex-specific sex-scores and weight, waist circumference, BMI, height, triglycerides, CRP, diastolic and systolic blood pressure (females only), and glucose, but not pulse (i.e., higher femaleness, lower trait values). For sum-scores, only HDL-cholesterol (females only) had a negative genetic correlation while all other traits were positively genetically correlated (females only for LDL-cholesterol; Fig. S5). Finally, regarding genetic correlations with sex-biased and cardiometabolic disorders, we identified that—within each sex—the sex-scores were negatively associated (i.e., higher femaleness, lower probability of these disorders) with type 1 diabetes, type 2 diabetes, rheumatoid arthritis, ischemic heart disease (females only), stroke (females only), ADHD, and depression (females only), and positively associated with anorexia (i.e., higher femaleness, higher probability of these disorders), all surviving a Bonferroni correction. A very similar pattern of effects was observed between the disorders and sum-scores, suggesting that these effects were driven by the aggregation of traits rather than latent “femaleness/maleness” (Fig. 5).

### Pleiotropy

Finally, we sought to evaluate and compare the pleiotropy of sex-score SNPs and sum-score SNPs. In females, 6001/27,622 (21.7%) sex-score SNPs and 1774/5678 (31.2%) sum-score SNPs were considered pleiotropic (associations with ≥ 8/12 constituent traits). Among the pleiotropic SNPs, 5081/6001 (84.7%) sex-score SNPs and 1545/1774 (87.1%) sum-score SNPs were considered concordant (same directionality of effects as the score in ≥ 2/3 nominally significant traits). For males, 3632/23,770 (15.3%) sex-score SNPs and 429/3049 (14.1%) sum-score SNPs were considered pleiotropic (≥ 8/12 traits). Among the pleiotropic SNPs, 3253/3632 (89.6%) sex-score SNPs and 183/429 (42.7%) sum-score SNPs were considered concordant (≥ 2/3; Fig. S6).

## Discussion

Here, we have elucidated the genetic architecture underlying our polyphenotypic sex-scores and sum-scores. We identified that while GWAS-identified sex-score genes were enriched for genes upregulated in the cervix and downregulated in brain tissues (particularly among females), sum-score genes were enriched for genes upregulated in the liver. Moreover, we identified “sex-different” SNPs along with female-dominant and male-dominant genes for both scores. Among these genes, the male-dominant genes were enriched for genes upregulated across multiple brain tissues while the female-dominant genes were enriched for genes expressed differentially in the hypothalamus, hippocampus, and cerebral cortex. There was also significant enrichment for three brain tissues among sum-scores in males, but no significant tissue enrichment for sum-scores in females. Finally, we identified genetic associations with sex-biased disorders and with cardiometabolic diseases, but these were largely similar for sex-scores and sum-scores, indicating that these genetic effects were driven by the aggregation of cardiometabolic traits, rather than latent “femaleness/maleness.”

The most striking functional differences between sex-score and sum-score GWASs emerged in the analyses of enrichment of gene expression across tissues. The sex-score genes, identified in females, were enriched for genes that were upregulated in the cervix and downregulated in the brain. Although this remains to be established, this effect is perhaps related to the actions of hormones and their receptors such as the oxytocin receptor, whose expression is critically modulated in both the brain^26^ and cervix^27^, and prevents masculinization in rodents^28^. This is also supported by our identification of significant hormone-related enrichment terms for sex-score genes but not sum-score genes. Moreover, the enrichment of sum-score genes (derived from cardiometabolic traits) in the liver may be related to its role in glucose and lipid metabolism, with consequences for cardiometabolic disorders such as type 2 diabetes, with which we demonstrated the sum-scores are genetically correlated^29^.

Furthermore, in our analyses of sdSNPs for each score, we found that male-dominant genes were enriched for genes upregulated in multiple brain tissues while female-dominant genes were enriched for genes differentially expressed in fewer brain tissues. Multiple brain regions control feeding behaviours, including those involved in homeostatic functions, which maintain energy balance (e.g., hypothalamus) and reward-related processing (e.g., basal ganglia, anterior cingulate cortex)^30–36^. Thus, the primarily male-dominant gene-enrichment in brain tissues may indicate a sex-biased pathway with potential relevance for effects on cardiometabolic traits. Indeed, there is evidence of sex differences in the hypothalamic regulation of homeostasis and feeding behaviours^37–39^. Additionally, differences between obese individuals and controls in “anatomical connectivity”, assessed with diffusion tensor imaging (DTI), have been reported in the basal ganglia with sex-specific effects^40^. Moreover, in a GTEx study of 29 tissues in humans, the most pronounced sex differences in brain tissues were in the basal ganglia^41^; in line with our male-dominant gene enrichment in the putamen, nucleus accumbens, and caudate, thus pointing to the possible sex bias in reward processing vis-à-vis effects on cardiometabolic syndrome.

Sex-score “maleness” was genetically correlated—in both males and females—with type 1 diabetes, type 2 diabetes, stroke, and ischemic heart disease, whereas sex-score “femaleness” was genetically correlated with anorexia; thus, higher “maleness” reflected cardiometabolic syndrome traits. We also found additional genetic associations between sex-score “maleness” and depression and ADHD; that is, traits not included in the sex-scores. The latter findings may nevertheless reflect indirect relationships between sex-scores and cardiometabolic syndrome, given that this syndrome is associated with depression^42^ and ADHD^43^, as well as rheumatoid arthritis^44^, and type 1 diabetes^45^. Given that the same pattern of effects was observed with sum-scores, these findings likely reflect the trait aggregation rather than latent “femaleness/maleness.”

Our finding that sex-scores and sum-scores were each highly genetically similar between the sexes is congruent with findings of a UK Biobank study that the majority of continuous traits are highly genetically correlated between the two sexes^16^. Moreover, in a previous study using sex-specific GWAS summary statistics across 20 behavioural traits, inter-sex genetic correlations approached r_g_ = 1, and only a few were significantly lower than r_g_ = 1, namely risk-taking and educational attainment^46^. To our knowledge, the most notable exception to this common pattern of genetic similarity between the sexes is testosterone, which demonstrates no genetic correlation and distinct effects between the sexes^47–50^.

Finally, we were interested in determining whether sex-score pleiotropic SNPs capture concordant “femaleness/maleness” across multiple traits. Among the pleiotropic sex-score SNPs, ~ 85–90% passed our directionality-concordance threshold, indicating that most pleiotropic sex-score SNPs capture “femaleness/maleness” across traits. In other words, we have identified a set of SNPs that are broadly implicated in “femaleness/maleness” rather than simply identifying a set of sex-score SNPs that are each associated with a single trait.

Here, we have examined the genetic architecture underlying polyphenotypic and polygenic sex-scores. Since these analyses are restricted to the UK Biobank, validation in external cohorts is warranted. While this trait is globally similar between the sexes with similar associated functions, distinct sex-specific effects at the level of single SNPs and tissue enrichments were identified. We have demonstrated how such scores partly reflect the summation of traits, but are phenotypically, genetically, and functionally distinct from these simple sums. Given the availability of increasingly large datasets with rich phenotypic, genetic, and gene expression data, quantitative and data-driven approaches to “femaleness/maleness” may be of high value, complementing gender-based studies of “femininity/masculinity”.

## Materials and methods

### Participants

The UK Biobank is a richly phenotyped and genotyped cohort comprising approximately 500,000 participants, recruited between 2006 and 2010 by 22 assessment centres in the United Kingdom^51^. Participants provided informed electronic signed consent, completed questionnaires and interviews, underwent functional and physical assessments, and provided blood, urine, and saliva samples^51^. All methods were carried out according to relevant guidelines and regulations^52^. The UK Biobank study was approved by the North West Multi-centre Research Ethics Committee as a Research Tissue Bank (see: https://www.ukbiobank.ac.uk/learn-more-about-uk-biobank/about-us/ethics). The study herein was approved under the UK Biobank Resource Application Number 43688 and by local ethics committees at the Research Institute of the Hospital for Sick Children (SickKids) and the Centre Hospitalier Universitaire (CHU) Sainte-Justine. The phenotypic assessments include physical measures, multimodal imaging, accelerometery, questionnaires, biochemical assays, and health outcomes^51^. The data (baseline measures only) were downloaded on March 12, 2020.

### Polyphenotypic sex-scores

Firstly, to render distributions across traits normal, positively skewed variables were log-transformed and values greater than or equal to 4 standard deviations from the mean were excluded as outliers. As previously conducted in the Saguenay Youth Study^17^, we created individual-level continuous sex-scores by summing standardized values across traits; for each trait, the standardized value was weighted by the respective sex-difference effect size (Table 1). This is described by the equation:

$$Sex\,\, score = \sum \left( {x_{i } \times B_{i} } \right),$$

in which $x$ indicates the participant's standardized value for each phenotype and $B$ indicates the sex effect size for each phenotype. The sex effect-sizes were derived using the semi-standardized beta coefficients corresponding to the effect of binary sex for each standardized trait, adjusting for age. We initially selected 13 routinely assessed anthropometric and cardiometabolic traits in the UK Biobank with large sample sizes (n ≥ 100,000) that were also available in other cohorts, including the Saguenay Youth Study (SYS), the Cardiovascular Health Study (CHS), the Framingham Heart Study (FHS) and the Rotterdam study (RS)^18–21^. To adjust for correlations among the comprising traits, pairs of traits with correlations exceeding r^2^ = 0.25, were averaged prior to computing sex-scores (Fig. S2), resulting in 9 traits. To facilitate interpretation and visualization, the sex-scores were normalized to achieve ranges between 0 and 1 as follows:

$$Normalized\,\, sex\,\, score = \frac{{Sex\,\, score - min\left( {Sex\,\, score} \right)}}{{max\left( {Sex\,\, score} \right) - min\left( {Sex\,\, score} \right)}},$$

with higher values signifying greater “femaleness.” Additionally, sum-scores were computed by summing up the values of the standardized 9 traits for each individual, *without* the sex-difference weighting. To avoid sample overlap, the GWAS sample was reserved for participants who passed genetic quality control (QC), described below, and who were not missing values on any of the comprising traits (n = 303,886). All other participants were used to compute the sex-difference effect sizes across the traits (n range: 123,731–195,880). Moreover, as a sensitivity analyses, we compared our approach of linear regression (i.e., Phenotype_i_ ~ Sex + Age) with logistic regression (i.e., Sex ~ Phenotype_i_ + Age). The coefficients extracted using linear regression and logistic regression were highly correlated (r = 0.97, r^2^ = 0.94, *p* = 2.02 × 10^–5^; Fig. S7). We decided to retain our original linear-model approach to estimating sex-difference effect sizes because although the coefficients were very similar for most of the traits (absolute difference ≤ 0.02 for 6/9 traits), differences emerged for traits with the largest effect sizes, particularly height (linear regression: − 1.40; logistic regression: − 2.79). Thus, we selected linear regression to minimize the overrepresentation of traits with the largest sex differences (Fig. S7). Additionally, as an external validation, we identified that the correlation between the sex-difference effect sizes among the UK Biobank and SYS adult participants were highly correlated (r = 0.94, *p* = 1.83 × 10^–6^; Fig. S8).

### Genome-wide association studies (GWAS)

To conduct GWAS analyses, we used PLINK 2.0^53^, assessing associations with sex-scores and sum-scores across single nucleotide polymorphisms (SNPs) in each sex. Before conducting association testing, the participants and SNPs were quality controlled (QC) in a sex-specific manner. We excluded individuals demonstrating heterozygosity or missingness outliers, a mismatch between genetic and reported sex, sex chromosomal aneuploidy, and non-European ancestry. Additionally, individuals with more than ten 3rd-degree relatives were removed, followed by the removal of individuals with close kinship using the R package ‘ukbtools’ version 0.11.3 (KING coefficient = 0.0884)^54^. We excluded SNPs with greater than 5% missingness, a minor allele frequency < 0.01, a significant deviation from Hardy Weinberg Equilibrium (threshold: *p* < 1 × 10^−10^), or an INFO score < 0.8. After the QC, the final “genetic” dataset included 209,383 females with 8,642,454 SNPs, and 181,389 males with 8,644,321 SNPs. Among these participants, there were 161,906 females and 141,980 males with values for sex-scores and sum-scores. We conducted sex-specific GWASs for the sex-score or sum-score as a dependent measure, implementing a general linear model, with age and the first 10 principal components of genetic ancestry as covariates.

In order to facilitate comparisons between the sex-specific GWASs, we created Miami plots using the R package, ‘miami plot’ (https://github.com/juliedwhite/miamiplot/). To map SNPs to genes, we used the functional mapping and annotation (FUMA)-GWAS platform^22^. Following the recommended parameters for positional mapping, we used an r^2^ ≥ 0.6 to define 'independent' significant SNPs, and an r^2^ ≥ 0.1 to define 'lead independent' significant SNPs. We used the reference panel population of 1000G Phase3 EUR, a minimum minor allele frequency (MAF) of 0.01, and a maximum distance of 250 kb between LD blocks, to constitute a locus. To perform positional mapping of SNPs to genes, FUMA searches for ‘candidate SNPs’ which are in LD (r^2^ ≥ 0.6) with the ‘independent SNPs’, and identifies genes within 10 kb of the either independent SNPs or candidate SNPs. To elucidate the functional roles of the identified genes, we used FUMA-GWAS's "GENE2FUNC" platform, inputting the list of genes mapped from SNPs, and testing their overrepresentation among genes from FUMA-GWAS's GWAS catalogue. We used the recommended parameters, namely a minimum of two overlapped genes and applying a false-discovery-rate Benjamini-Hochberg (FDR-BH) correction for multiple comparisons.

### Genetic correlations

Firstly, we conducted an inter-sex genetic correlation between the sex-specific GWASs for sex-scores. To assess whether the inter-sex genetic correlation differed from 1, we used the equation, ${\text{z }} = { }\frac{{1{ } - { }r_{{\text{g}}} }}{SE}{ }$. Secondly, we conducted genetic correlations between the sex-specific sex-score and sum-score GWASs and the sex-specific traits that comprised them. Thirdly, we conducted genetic correlations between the sex-specific sex-score and sum-score GWASs and previously published GWASs for sex-biased disorders and metabolic-syndrome disorders. Based on sex differences in prevalence^8,55^, the sex-biased disorder GWASs comprised autoimmune disorders (systemic lupus erythematosus, rheumatoid arthritis, multiple sclerosis, type 1 diabetes), psychiatric disorders (anorexia, anxiety, substance abuse, autism, attention deficit hyperactivity disorder [ADHD], depression), and inflammatory bowel syndrome. Moreover, given the inclusion of anthropometric and cardiometabolic traits in the sex-scores, we also assessed genetic correlations with type 2 diabetes, ischemic heart-disease, and stroke. Information about the sources of these summary GWAS statistics is provided in Table S6. These analyses were run using linkage disequilibrium score regression (LDSC) version 1.0.1 (https://github.com/bulik/ldsc/wiki/Heritability-and-Genetic-Correlation)^24,25^. The analyses were restricted to HapMap3 SNPs and we used LDSC's 1000 Genomes European LD scores (https://data.broadinstitute.org/alkesgroup/LDSCORE/). Bonferroni corrections were applied to the genetic correlation analyses, for the traits comprising each of the scores (13 traits × 2 sexes × 2 scores = 52 tests; p < 0.00096) and the clinical conditions (15 conditions × 2 sexes × 2 scores = 60 tests; p < 0.00083).

### Sex-different single nucleotide polymorphisms (sdSNPS)

To compute sdSNPs, we used the following equation:

$$t = \frac{{B_{males} - B_{females} }}{{\sqrt {SE_{ males}^{ 2} + SE _{females }^{ 2} - 2r \times SE _{{\begin{array}{*{20}c} {males } \\ \\ \end{array} }} \times SE_{{\begin{array}{*{20}c} { females } \\ \\ \end{array} }} } }}$$

whereby *B* indicates the standardized beta weight for each SNP, *SE* indicates the standard error for each SNP, and *r* indicates the overall inter-sex Spearman’s correlation between all the effects of all the retained SNPs^16,56^. For sex-scores and sum-scores, we retained SNPs that were nominally (p < 0.05) in at least one sex. We excluded SNPs that were associated with sex as a dependent variable, as associations with these SNPs likely resulted from sex-specific participation bias^57^, leaving 1,844,503 and 1,426,959 SNPs for sex-scores and sum-scores, respectively. We considered SNPs “male-dominant” if the absolute beta coefficient was greater in males than females (abs[*B*_males_] > abs[*B*_females_]), and “female-dominant’ for the opposite effect (abs[*B*_females_] > abs[*B*_males_])^56^. Following the example of Bernabeu et al., two-tailed p-values were transformed to one-tailed p-values, such that the p-value list for males (${\text{p}}_{M} )$ was computed as ${\text{p}}_{M} = \frac{{{\text{p}}_{2T} }}{2}$ for “male-dominant” SNPs, and ${\text{p}}_{M} = 1 - \left( {\frac{{{\text{p}}_{2T} }}{2}} \right)$ for female-dominant SNPs^16^. Similarly, the p-value list for females $\left( {{\text{p}}_{F} } \right)$ was computed as ${\text{p}}_{F} = \frac{{{\text{p}}_{2T} }}{2}$ for “female-dominant” SNPs, and ${\text{p}}_{F} = 1 - \left( {\frac{{{\text{p}}_{2T} }}{2}} \right)$ for male-dominant SNPs.

Subsequently, we inputted the full lists of male SNPs and female SNPs with one-tailed p-values, separately, on the FUMA-GWAS platform. Using this platform, we performed a gene-wide association analysis (MAGMA) to retrieve p-values for each gene. Finally, we conducted gene enrichment analyses using FUMA’s “GENE2FUNC”. Analyses and data preparation were conducted using R version 4.1.1^58^, including the R packages ‘tidyverse’ version 1.14.2^59^, ‘data.table’ version 1.3.1^60^ and ‘broom’ version 0.8.0 (https://CRAN.R-project.org/package=broom).

### Pleiotropy

For each of the GWAS-significant sex-score SNPs, we counted the number of nominally significant associations with the 12 constituent traits and set our pleiotropy threshold at 8/12. We then counted the degree of concordance in the directionality of the effect of each sex-score SNP and the directionality of each constituent trait and set our concordance threshold at 2/3. For sex-scores, concordance was based on the sex-difference effect size. For example, a SNP was considered concordant between sex-scores and HDL if both effects were positive since a higher sex-score indicates greater “femaleness” and HDL is higher in females, compared with males. These analyses were repeated for the sum-scores for comparison.

## Supplementary Information

Supplementary Information 1.

Supplementary Information 2.

Supplementary Information 3.

Supplementary Figures.

## Data Availability

The data can be provided by the UK Biobank pending scientific review and a completed material transfer agreement. Applications for access to the data can be completed at: https://www.ukbiobank.ac.uk/enable-your-research/apply-for-access. Data base produced during this study is also available from corresponding author on reasonable request. GWAS summary statistics are available on the GWAS Catalog (https://www.ebi.ac.uk/gwas/) under the following study accession IDs: GCST90270116, GCST90270117, GCST90270118, and GCST90270119. Finally, PLINK and R scripts have been provided as supplemental files.
