# Supplementary material for: The genetics of a “femaleness/maleness” score in cardiometabolic traits in the UK biobank
Source: Sci Rep. 2023 Jun 5;13:9109. doi: 10.1038/s41598-023-36132-1 (PMC10241870; doi:10.1038/s41598-023-36132-1)
Supplement: Supplementary file 4 — Supplementary Figures. [file 41598_2023_36132_MOESM4_ESM.pdf]

Title: The Genetics of a “Femaleness/Maleness” Score in Cardiometabolic Traits in the UK Biobank

Authors: Daniel E. Vosberg, Ph.D., Zdenka Pausova, M.D., *Tomáš Paus, M.D./Ph.D.

Supplementary Figures

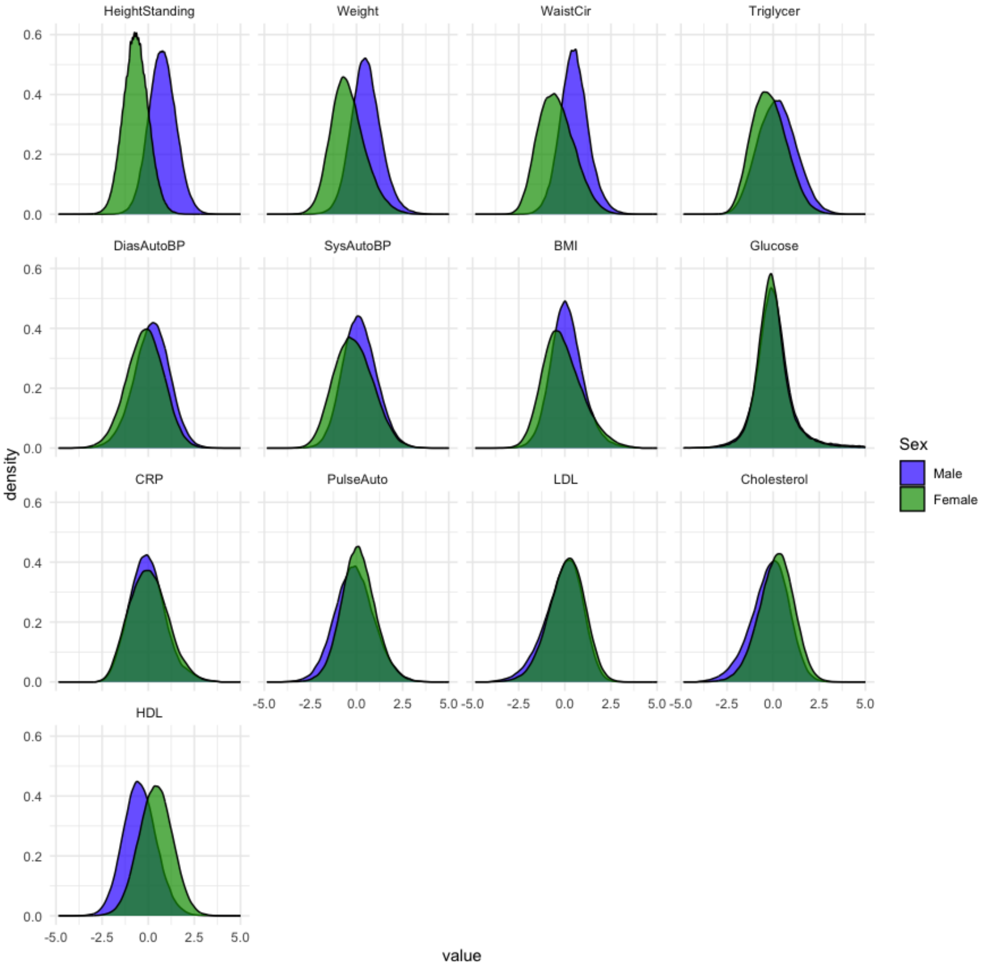

Figure S1. Density plot distributions of the age-adjusted traits comprising the sex-scores and sum-scores.

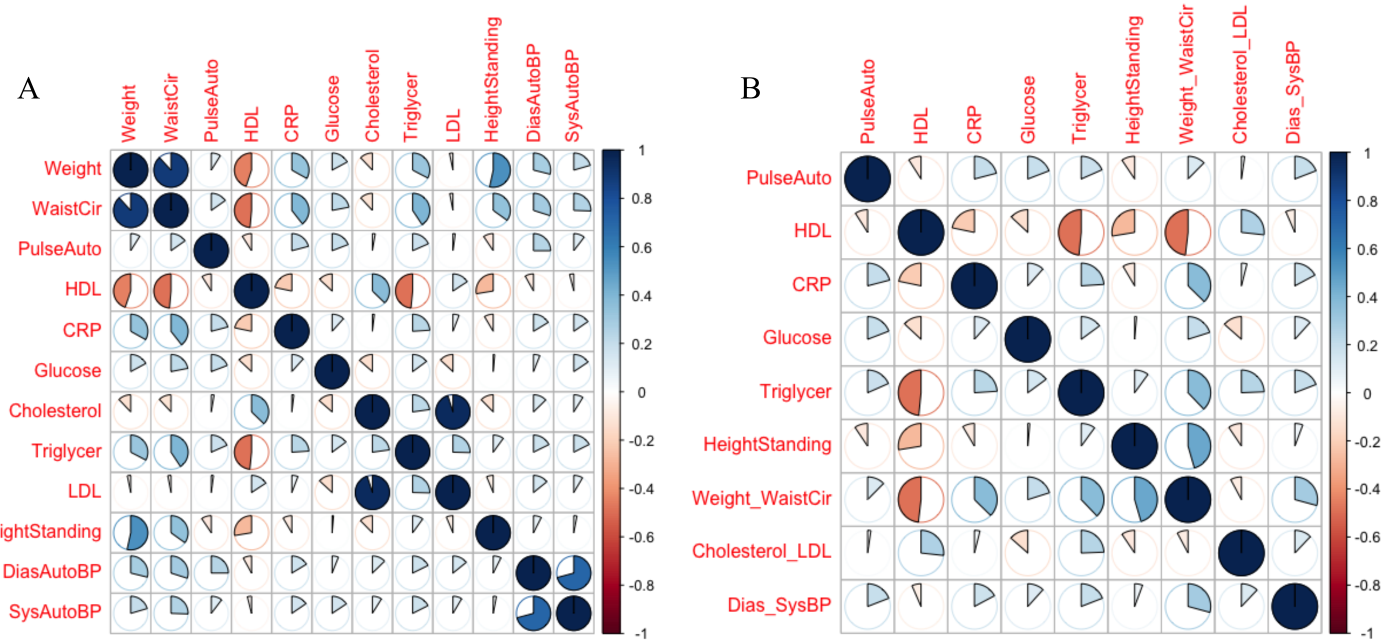

Figure S2. Correlations among composite traits before and after averaging. We considered the correlations among the comprising traits by averaging pairs of traits that were moderately correlated (r ≥ 0.5, r2 ≥ 0.25; Figure A). We thus averaged (1) weight and waist circumference, (2) diastolic and systolic blood pressure, and (3) cholesterol and LDL. We also removed body mass index (BMI) since BMI is mathematically related to both weight and height. The resulting correlation matrix among the 9 traits is presented below (all $r < 0.5$; $r^2 < 0.25$; Figure B). The pie charts indicate the strength and direction of the correlations, with values ranging from r = -1 (filled circle; dark red) to r = 0 (empty circle; white) to r = 1 (filled circle; dark blue).

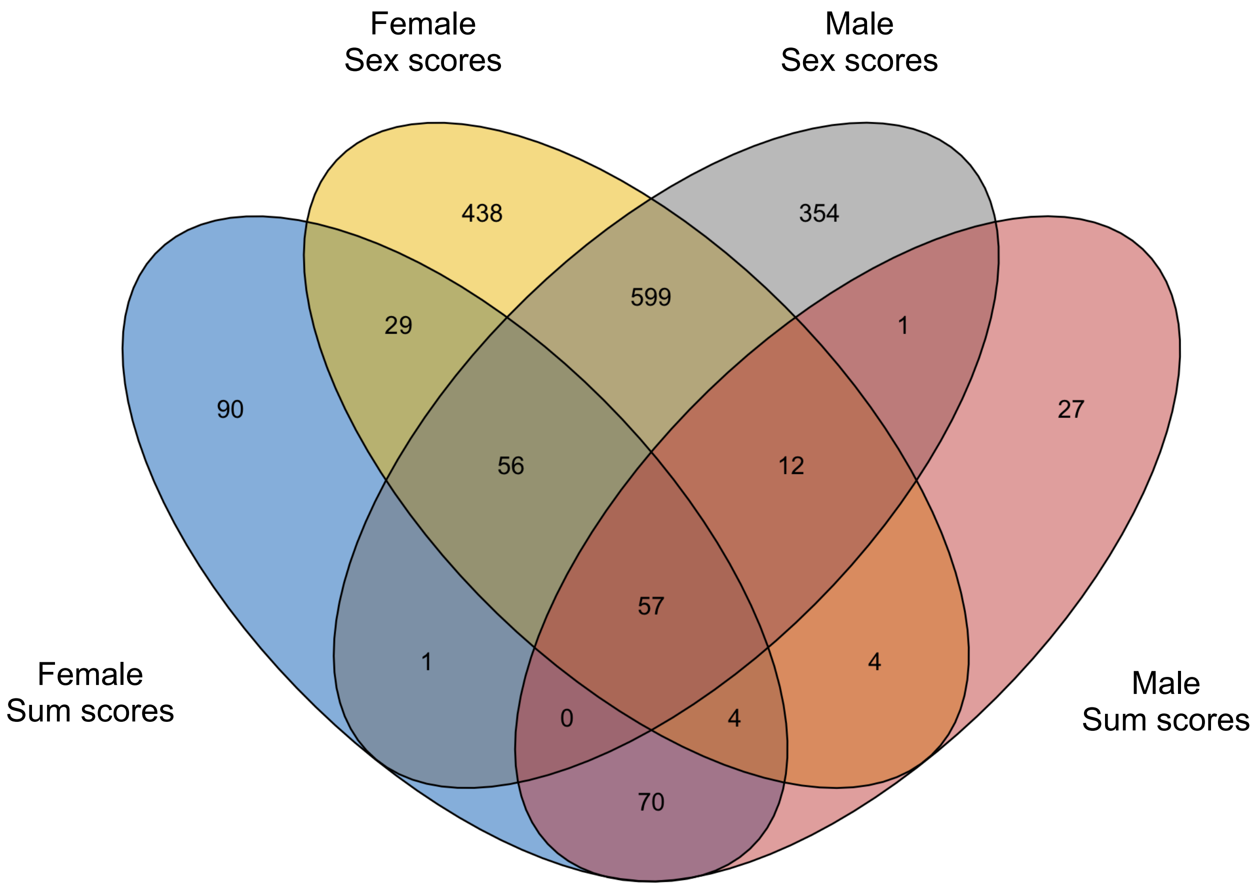

Figure S3. Venn diagram of genes mapped using FUMA-GWAS from the sex-specific sex-score and sum-score GWASs. The gene numbers represent Entrez IDs with female sex-scores in yellow, female sum-scores in blue, male sex-scores in grey, and male sum-scores in pink. These gene lists were subsequently inputted into the ‘compareCluster’ gene enrichment analyses presented and described in Figure S4.

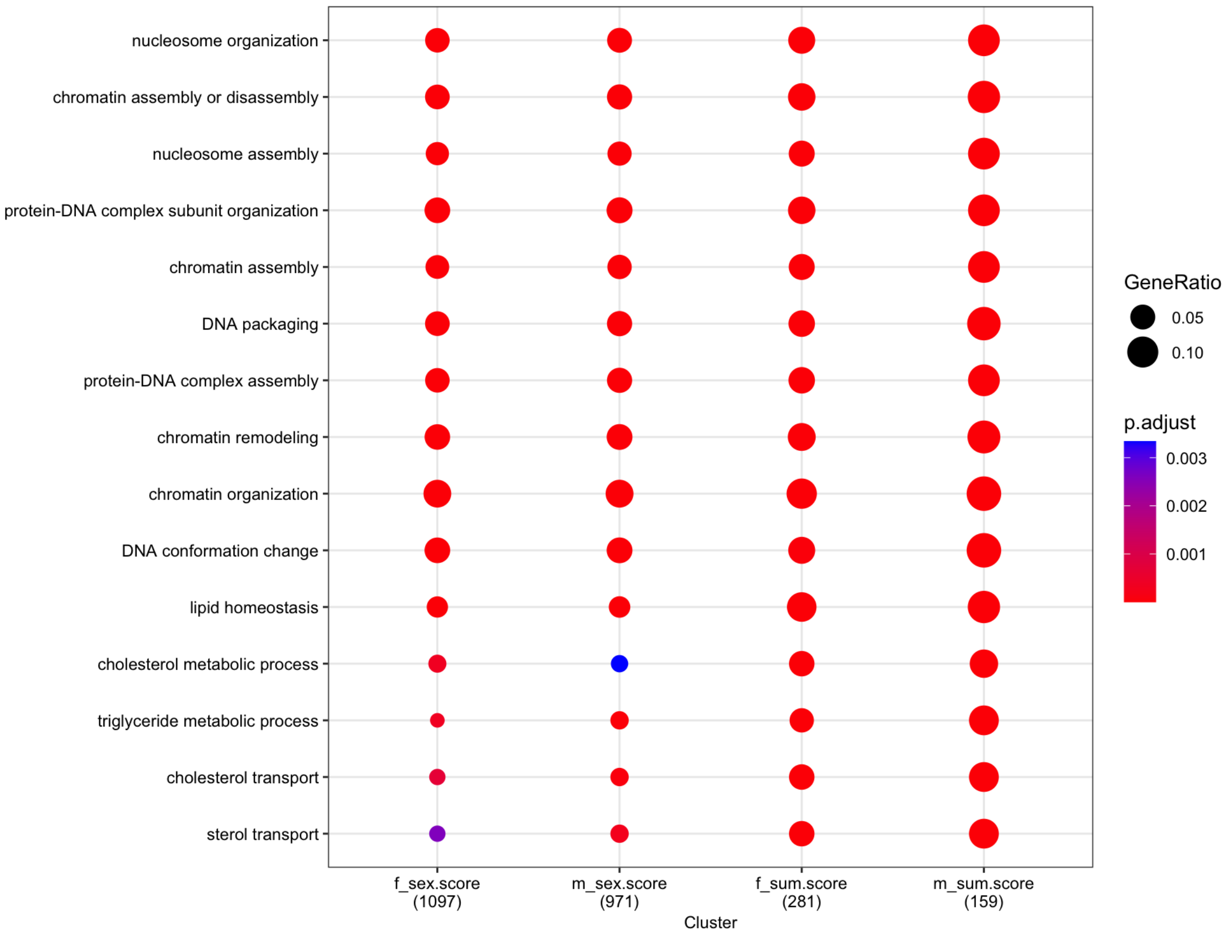

Figure S4. Results of gene enrichment analyses of biological processes the sex-specific sex-score and sum-score GWAS genes, mapped using FUMA-GWAS. These Entrez ID gene numbers are listed below for each GWAS gene list. We used the 'compareCluster' function of the R package, 'clusterProfiler', using 'enrichGO' to conduct gene ontology of biological processes and compare the results. The default settings were used, namely a minimum gene set size of 10 and maximum gene set size of 500, and a Benjamini-Hochberg (BH) adjustment for multiple comparisons. The size of circles represents the gene ratio enrichment and color represents the adjusted p-value. The top 10 significant GO terms are presented for each gene list, with comparisons across lists, identifying great similarity and subtle differences only in the magnitudes of enrichment.

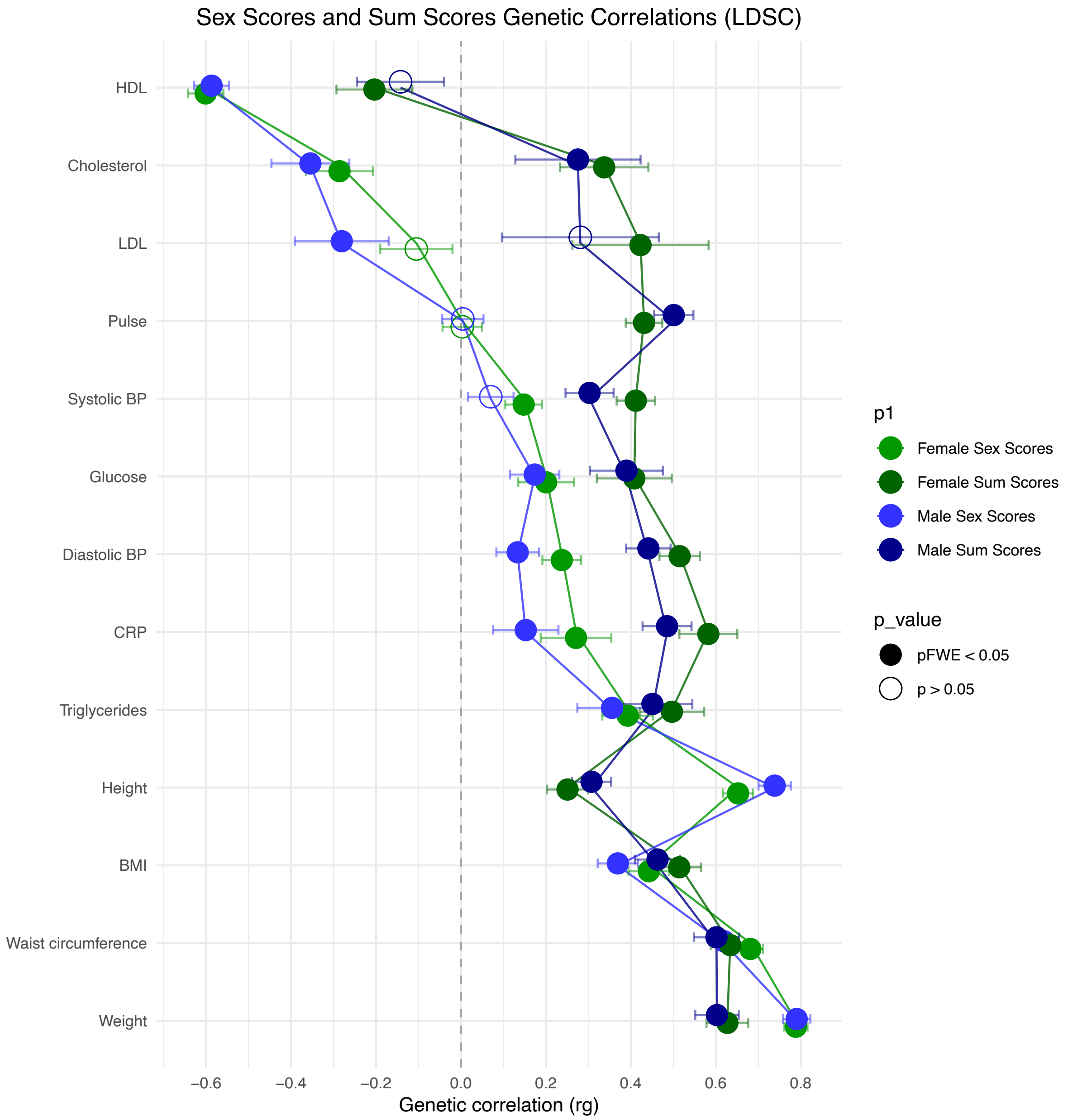

Figure S5. Genetic correlations of the sex-scores and sum-scores with comprising traits. To facilitate comparisons between scores, the sign of sex-scores was flipped. Generated using linkage disequilibrium score regression (LDSC), sex-stratified genetic correlations were conducted between the sex-score GWASs and sex-specific traits that comprise them. The significant effects are filled-in, surviving a Bonferroni correction for 52 genetic correlations for each score (13 traits x 2 sexes x 2 scores; $p < 0.00096$). The error bars represent the 95% confidence intervals.

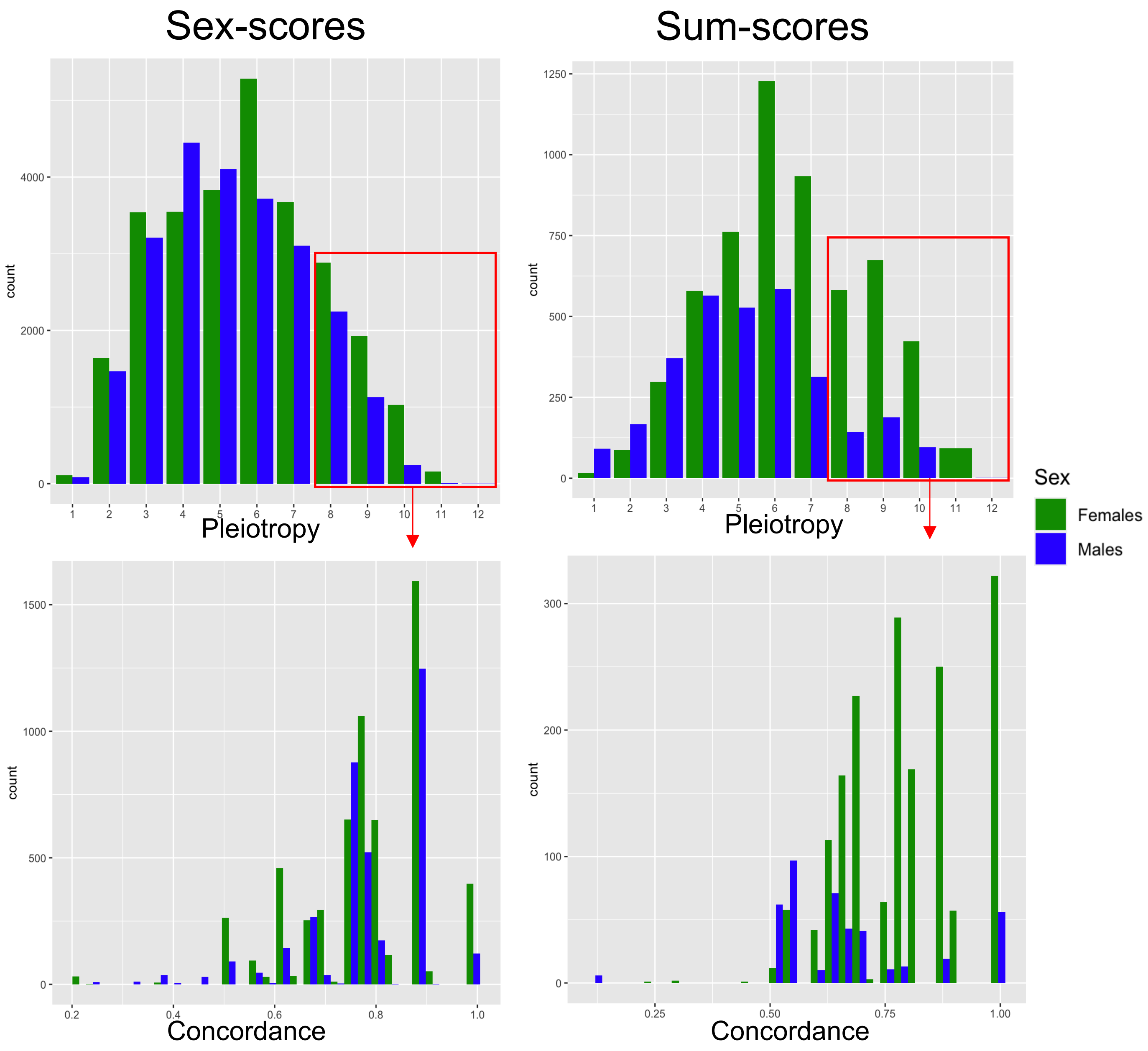

Figure S6. Distributions of the degree of pleiotropy and concordance among GWAS-sig. SNPs. Among pre-clumped GWAS-significant sex-score (top-left) and sum-score SNPs (top-right), the number of nominally significant associations with the 12 constituent traits is shown. In the bottom row, among the more pleiotropic SNPs (>= 8/12 nominally significant trait associations; red boxes) for each score, the fraction of traits with betas matching the directionality of each score is shown.

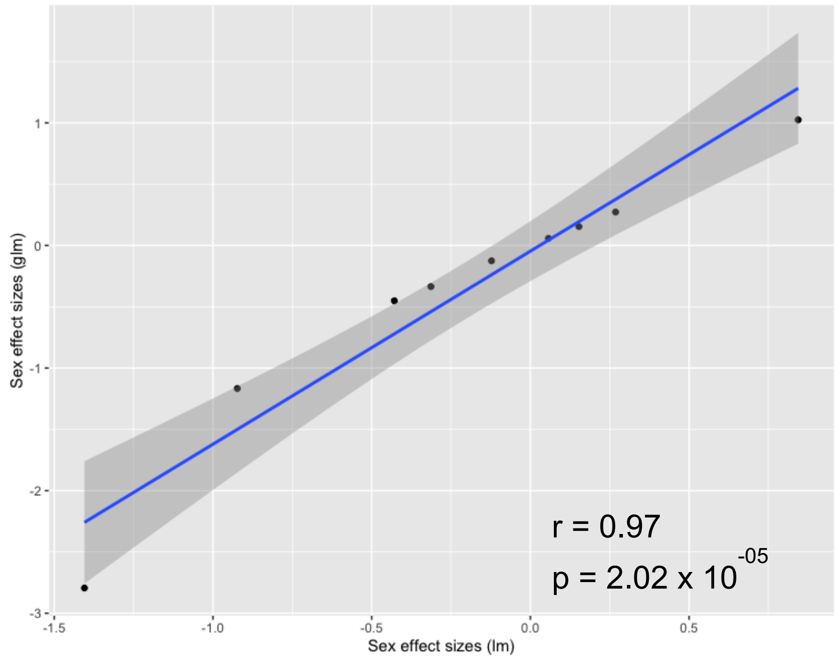

Variable	Linear (Beta)	Logistic (Beta)	Difference
Height	-1.4	-2.79	1.39
Weight & Waist Circumference (averaged)	-0.923	-1.17	0.247
Triglycerides	-0.429	-0.451	0.022
Diastolic & Systolic BP (averaged)	-0.313	-0.336	0.023
Glucose	-0.122	-0.125	0.003
CRP	0.057	0.0579	-0.0009
Pulse rate	0.153	0.154	-0.001
Cholesterol and LDL (averaged)	0.269	0.272	-0.003
HDL	0.844	1.02	-0.176

Figure S7. Comparison of effect sizes computed using linear regression compared to those computed using logistic regression. While the correlation between the effect sizes for each approach is high, differences emerge for variables with the largest effect sizes, particularly height. In order to avoid disproportionately dominating the sex-score by height, we selected linear regression to compute our sex-difference weightings.

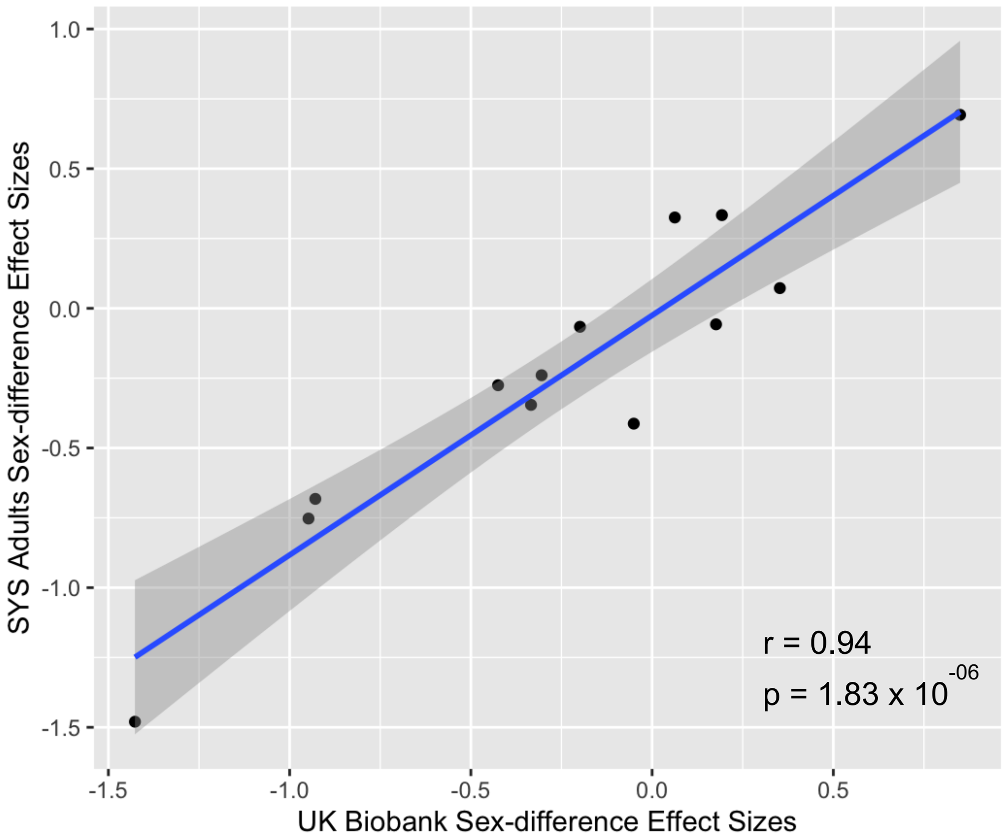

Figure S8. Correlation between the sex-difference effect sizes in the UK Biobank and the Saguenay Youth Study (SYS) adults. The between-cohort effect sizes were highly correlated (r = 0.94, p = 1.83 x 10^{-6}).
